# Nonequilibrium Photocarrier and Phonon Dynamics in Dirac Semimetal NiTe_2_ Microcrystals Probed by Ultrafast Reflectivity Spectroscopy

**DOI:** 10.3390/nano16030204

**Published:** 2026-02-05

**Authors:** Shijie Ma, Kaiwen Sun, Peng Suo, Guohong Ma

**Affiliations:** Department of Physics, Shanghai University, Shanghai 200444, China; ma123@shu.edu.cn (S.M.);

**Keywords:** 3D Dirac semimetal, transient reflectance spectroscopy, ultrafast dynamics, electron-optical phonon coupling

## Abstract

Topological 3D Dirac semimetals are characterized by bulk Dirac cone band crossings and nontrivial topological surface states, giving rise to a wealth of exotic physical properties and attracting considerable attention in recent years. Understanding the nonequilibrium dynamics of Dirac semimetals in micro-size provides critical guidance for the design of micro- and nanoscale optoelectronic and ultrafast photonic devices. In this work, we employ time-resolved microscopic transient spectroscopy to investigate the nonequilibrium photocarrier and lattice dynamics in microcrystalline Dirac semimetal NiTe_2_, a prototypical 3D Dirac semimetal. Following photoexcitation at 390 nm, the transient reflectivity kinetics of NiTe_2_ can be well described with a triple-exponential decay function. The fastest relaxation component occurs on a sub-picosecond timescale and increases with pump fluence, which originates from electron-optical phonon coupling. An intermediate relaxation process with a characteristic time of ~8 ps is attributed to electron–hole recombination, while a slower decay component on the order of ~20–30 ps can be assigned to the anharmonic decay of optical phonons into acoustic phonons. Polarization-resolved measurements reveal nearly in-plane isotropic transient responses, which are insensitive to the polarization of probe light. These findings contribute to the physical insights for the development of future photonics and optoelectronic devices based on topological Dirac semimetals.

## 1. Introduction

Three-dimensional (3D) topological Dirac semimetals are a prototypical class of quantum materials in which conduction and valence bands cross linearly at discrete Dirac points, giving rise to massless Dirac fermions characterized by linear energy-momentum dispersion [1,2,3,4]. With their symmetry-protected band crossings [5,6], nontrivial topological surface states [7,8], and strong spin–orbit coupling [9,10], 3D Dirac semimetals have emerged as a versatile platform for investigating relativistic quasiparticles and various exotic quantum phenomena in condensed matter systems. Beyond fundamental significance, they also hold great promise for next-generation photonics [11], optoelectronic [12], and terahertz technologies [13], where their low energy dissipation and ultrahigh carrier mobility play a crucial role.

Among the family of 3D Dirac semimetals, NiTe_2_ has recently been identified as a type-II Dirac semimetal, characterized by strongly tilted Dirac cones [14,15]. More importantly, its Dirac nodes are located close to the Fermi level [16], meaning that the resulting Dirac fermions participate in the charge transport, thus providing an ideal platform for investigating the transport behaviors of type-II Dirac Fermions. An experimental study has demonstrated the planar Hall effect arising from the chiral anomaly of topological electrons in NiTe_2_ [17]. In addition, strong spin–orbit coupling combined with lattice anharmonicity in NiTe_2_ leads to complex electro-phonon and phonon-phonon interactions, as revealed by Raman spectroscopy and supported by first-principles calculations [18]. Furthermore, NiTe_2_ has also emerged as a promising candidate for optoelectronic devices [12], particularly in high-performance photodetectors [19,20].

Ultrafast spectroscopy has served as a powerful tool for elucidating nonequilibrium carrier and lattice dynamics in 3D Dirac semimetals [21]. To date, extensive transient studies have been carried out on representative 3D Dirac semimetals, such as Cd_3_As_2_ [22,23], PdTe_2_ [13,24], and PtTe_2_ [25,26], uncovering the fundamental dynamical evolution of microscopic degrees of freedom and clarifying the underlying interaction mechanism among various elementary excitations. Carrier relaxation dynamics are highly sensitive to the electronic structure and scattering mechanisms in a material. Thus, a profound understanding of the ultrafast carrier relaxation behaviors in NiTe_2_ may offer valuable insights into the connection between the macroscopic physical properties and their microscopic origins.

A recent study has reported carrier relaxation and coherent phonon dynamics in bulk single-crystalline NiTe_2_, thereby providing a basic understanding of its photoexcited nonequilibrium response [27]. Nevertheless, the transient dynamics in high-quality NiTe_2_ microcrystal flakes remain largely unexplored. In fact, micrometer-sized flakes are more directly relevant to practical device architectures, as integrated optoelectronic and photonic devices are typically fabricated from micro- or nanoscale materials rather than bulk crystals. Consequently, investigating ultrafast carrier dynamics at the microcrystal scale is essential for the physics-guided design and optimization of NiTe_2_-based integrated devices.

In this work, we investigate the ultrafast nonequilibrium dynamics of NiTe_2_ microcrystals deposited on a sapphire substrate using optical pump–optical probe (OPOP) spectroscopy. The temporal evolution, encompassing initial electronic excitation, energy transfer to the lattice, and eventual thermal diffusion, is explicitly resolved. Upon 390 nm photoexcitation, NiTe_2_ displays a sub-picosecond recovery component governed by electron–phonon (e-ph) coupling, followed by a secondary relaxation process on a timescale of ~8 ps arising from electron–hole (e-h) recombination. A slower relaxation component is attributed to the anharmonic decay of hot optical phonons into cold acoustic phonons, while the residual long-lived offset is associated with heat diffusion. Furthermore, polarization-dependent kinetics show that the ultrafast optical response is nearly in-plane isotropic and insensitive to the probe polarization. These results elucidate the relaxation pathways of ultrafast carrier and phonon dynamics in 3D Dirac semimetal NiTe_2_ microcrystals, validating their suitability for application in microscale integrated ultrafast optoelectronic devices.

## 2. Experimental Setup and Characterizations

The nonequilibrium ultrafast photocarrier dynamics of NiTe_2_ microcrystals were investigated using a home-built micro-area pump-probe system in a reflection geometry. The laser pulses were delivered from a Ti: sapphire regenerative amplifier (Spitfire Pro, Spectra-Physics, Milpitas, CA, USA) operating at a center wavelength of 780 nm, a pulse duration of 120 fs, and a repetition rate of 1 kHz. The output beam was divided into two branches: one served as the probe beam at the fundamental wavelength (780 nm), while the other beam was frequency-doubled using a β-BBO crystal to generate 390 nm (3.18 eV) light pulses for ultrafast pumping. The transient reflectivity signals were collected via a lock-in amplifier (SR830, Stanford Research Systems, Sunnyvale, CA, USA). All measurements were performed at room temperature under ambient conditions.

**Figure 1:**
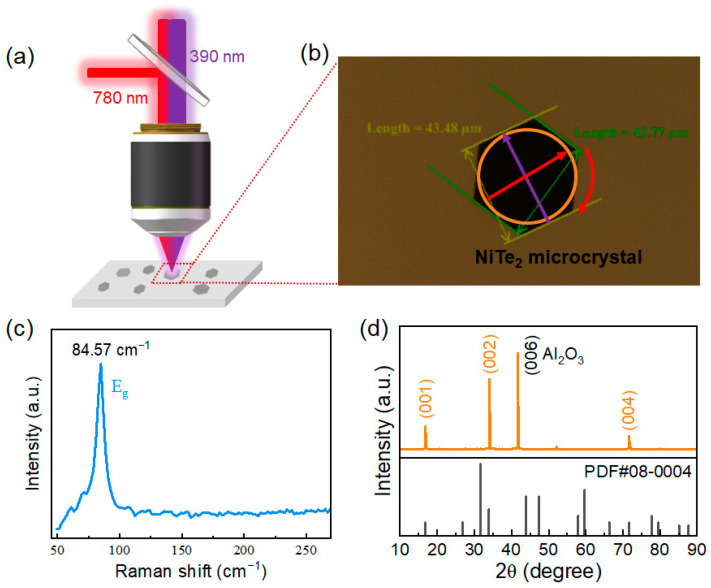
Micro-area OPOP setup and structural characterization of NiTe_2_ microcrystals. (**a**) Schematic illustration of the OPOP experimental system with a reflective configuration. A 390 nm pump beam (purple) and a 780 nm probe beam (red) are collinearly focused through a microscope objective onto a single NiTe_2_ flake grown on a sapphire substrate. The reflected probe beam is collected and guided to the detector. (**b**) Optical image of the hexagonal NiTe_2_ microcrystal shown in (**a**). The brown-yellow and green lines mark the characteristic lateral dimensions of the NiTe_2_ microcrystal, while the orange-shaded circular region indicates the spatial area excited by the pump beam. The purple and red arrows denote the polarization directions of the pump and probe beams, respectively, which are mutually orthogonal. During polarization-dependent measurements, the probe polarization is rotated, as indicated by the red arrow. (**c**) Raman spectrum of the NiTe_2_ microcrystal, showing a prominent characteristic peak at 84.57 cm^−1^ corresponding to the E_g_ phonon mode. (**d**) X-ray diffraction (XRD) pattern of the NiTe_2_ microcrystal. The diffraction peaks at 16.74°, 33.87°, and 71.72° are indexed to the (001), (002), and (004) crystal planes of NiTe_2_, respectively. The additional peak at 41.75° originates from the sapphire substrate.

A schematic illustration of the experimental setup is shown in Figure 1a. The 390 nm pump beam (purple) and the 780 nm probe beam (red) were collinearly focused onto a selected NiTe_2_ microcrystal through a high-numerical-aperture objective lens. An optical image of a representative NiTe_2_ microcrystal is presented in Figure 1b, revealing a well-defined hexagonal morphology with an edge length of ~43 μm. The pump and probe spots had diameters of approximately 40 μm and 30 μm, respectively, with spatial overlap on the sample. The polarizations of the pump and probe beams were set to be orthogonal, as indicated in Figure 1b. During polarization-dependent measurements, the probe polarization was rotated with respect to the fixed pump polarization (denoted by the curved red arrow), with 0° corresponding to the orthogonal configuration.

NiTe_2_ microcrystals were synthesized directly on sapphire substrates (1 cm × 1 cm) via chemical vapor deposition. The microcrystals have a thickness of ~100 nm, and Figure 1a illustrates the spatial distribution of isolated microcrystal flakes on the sapphire substrate. Raman spectroscopy measurements were carried out under 532 nm excitation, with the spectrum in the range of 50–250 cm^−1^ displayed in Figure 1c. A characteristic peak at ~84.6 cm^−1^, corresponding to the in-plane E_g_ vibrational mode, is clearly resolved, in good agreement with previous reports [28,29]. X-ray diffraction (XRD) measurements were further performed, as shown in Figure 1d. Sharp diffraction peaks indexed to the (001), (002), and (004) planes are observed, indicating the high crystallinity and a preferential orientation along the c-axis [29,30]. An additional diffraction peak at 41.8° originates from the sapphire substrate. These structural characterizations confirm the high crystalline quality of the investigated NiTe_2_ microcrystals.

## 3. Results and Discussion

**Figure 2:**
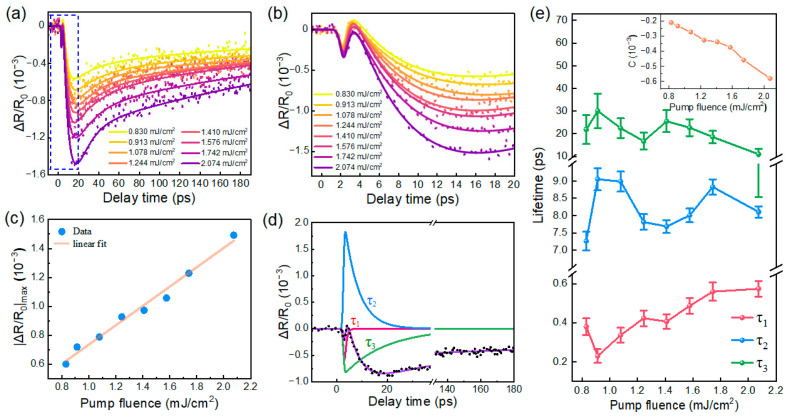
**Pump fluence dependence of the ultrafast reflection response in NiTe_2_ microcrystals.** (**a**) Transient reflectivity traces measured at different pump fluences. The colored data points represent the experimentally measured Δ*R*/*R*_0_ signals, and the corresponding solid lines are tri-exponential fitting results. (**b**) Enlarged view of the transient signals within the first 20 ps. (**c**) Δ*R*/*R*_0_ peak amplitude as a function of pump fluence. The solid line represents a linear fit. (**d**) Representative fitting result at a selected pump fluence, displaying the experimental data (colored dots), the overall fit (purple curve), and the decomposed relaxation components (*τ*_1_: red, *τ*_2_: blue, *τ*_3_: green). (**e**) Extracted relaxation lifetimes with respect to pump fluence. The inset shows the offset term C versus pump fluence.

The time-resolved differential reflectance, Δ*R*/*R*_0_, was recorded by varying the delay time between the pump and probe pulses, where *R*_0_ denotes the optical reflection of the probe light in the absence of photoexcitation, and Δ*R* represents the photoinduced change in reflection of the probe beam. Figure 2a displays the transient reflection spectra of NiTe_2_ measured at different pump fluences, with Figure 2b providing an enlarged view of the first 20 ps (dashed box in Figure 2a). Notably, the relaxation kinetics exhibit multiple distinct stages. Following photoexcitation, the Δ*R*/*R*_0_ response exhibits a sharp drop to a minimum, followed by a rapid recovery, with the signal slightly rising above zero at low pump fluences. Subsequently, the Δ*R*/*R*_0_ decreases again, reaching a second minimum at approximately 16 ps, and then gradually relaxes back to the equilibrium state over a prolonged timescale extending to several hundreds of picoseconds. The peak intensity, i.e., |Δ*R*/*R*_0_|_max_, is plotted in Figure 2c, revealing a linear dependence with respect to pump fluence. This behavior indicates that the transient response does not saturate within the pump fluence range employed in this work.

To quantitatively extract the relaxation lifetimes and disentangle the complicated relaxation processes, a tri-exponential decay function convoluted with the laser pulse width in the following form was tentatively used to fit the transient reflectivity traces:

(1) $$\frac{\Delta R}{R_{0}}=\sum_{i=1,2,3}A_{i}\;\exp\left(\frac{-t}{\tau_{i}}\right)\;\cdot \;\mathrm{erfc}\left(\frac{\omega }{\tau_{i}}-\frac{t}{2\omega }\right)+C$$

where A*_i_* and *τ_i_* represent the amplitude and decay time of the *i*-th relaxation component, respectively. *t* is the delay time between the pump and probe pulses, ω is the temporal duration of the laser pulse, C denotes a constant offset, and erfc(*t*) = 1 − erf(*t*) is the complementary error function. As shown in Figure 2a (solid line), the measured Δ*R*/*R*_0_ response can be well reproduced by the fitting model, yielding three distinct characteristic time constants as well as a negative offset. Figure 2d shows a representative fitting result decomposed into three dominant components together with the total fitted curve at a selected pump fluence. Among these components, the *τ*_2_ process contributes positively to Δ*R*/*R*_0_ (i.e., A_2_ > 0), which is attributed to photoinduced bleaching associated with conduction-band filling, that is, the population of pump-excited electrons in the conduction band suppresses interband transitions at probing wavelength through Pauli blocking. In contrast, the *τ*_1_, *τ*_3_, and offset components contribute negatively to Δ*R*/*R*_0_ (i.e., A_1_, A_3_, C < 0), and are related to thermally driven processes. The extracted relaxation lifetimes as a function of pump fluence are summarized in Figure 2e, with the fitted constant C shown in the inset. Below, we analyze the underlying microscopic mechanisms responsible for the observed ultrafast reflectance dynamics step by step.

For the semimetal NiTe_2_, optical excitation with 390 nm pump pulses promotes a large population of electrons into the conduction band, leaving holes in the valence band. The photoexcited electron system rapidly thermalizes via electron-electron (e-e) scattering, forming a quasi-equilibrium Fermi–Dirac distribution characterized by an elevated electronic temperature relative to the lattice temperature owing to the different heat capacities of electrons and phonons. Subsequently, the relaxation dynamics are generally dominated by the cooling of hot electrons through electron-optical phonon scattering, during which excess electronic energy is transferred to the lattice system. This process typically occurs on a sub-picosecond to few-picosecond timescale and can be described within the framework of the two-temperature model (TTM) [31,32,33]. It is worth noting that the TTM is commonly employed to interpret the cooling and relaxation dynamics of hot electrons in metallic or semimetallic systems [34,35,36].

**Figure 3:**
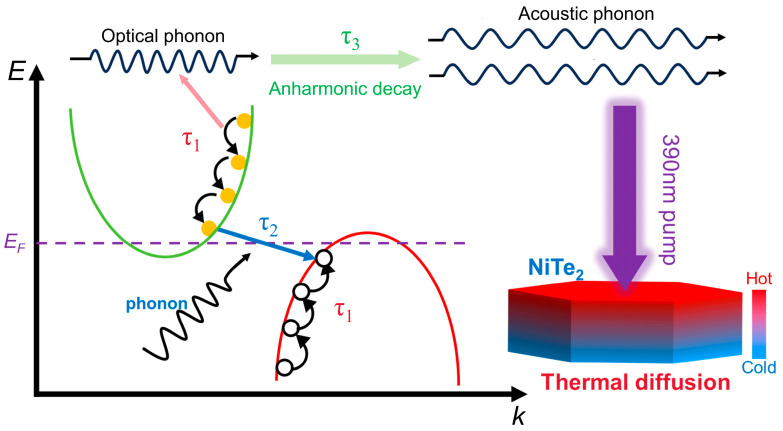
Schematic illustration of ultrafast carrier and phonon relaxation dynamics in NiTe_2_ microcrystals. The red and green curves represent the valence band and conduction band, respectively, while the purple dashed line denotes the Fermi level. The purple arrow indicates photoexcitation by the 390 nm pump pulse. The blue arrows illustrate the phonon-assisted electron–hole recombination process corresponding to *τ*_2_. The pink arrows represent optical phonons involved in electron–phonon coupling (*τ*_1_). The light-green arrows depict the anharmonic decay of optical phonons into acoustic phonons (*τ*_3_). The subsequent thermal diffusion process in the microcrystal after photoexcitation is illustrated using red (hot) and blue (cold) color contrast.

In our NiTe_2_ microcrystals, the fastest relaxation component, *τ*_1_, occurs on a sub-picosecond timescale and increases with pump fluence. Both its timescale and fluence dependence are consistent with the TTM, in which the e-ph interaction time can be expressed as [37,38]:

$${1/\tau }_{e\text{-}\mathrm{ph}}=3\hbar \lambda \langle \omega^{2}\rangle /\pi k_{B}T_{e}$$

where *λ*<ω^2^> is the second moment of the Eliashberg function, *k*_B_ is the Boltzmann constant, and *T*_e_ is the electronic temperature. Within this scenario, a higher pump fluence leads to a higher electronic temperature *T*_e_, thereby leading to a longer e-ph coupling time *τ*_e-ph_. Accordingly, we ascribe the fastest relaxation process *τ*_1_ to the cooling of the hot electrons that relax toward the conduction band edge via e-ph interaction, similar to observations in other semimetal materials [34,39,40]. A schematic illustration of the relaxation picture in NiTe_2_ microcrystals is presented in Figure 3, which will help us to understand the origin of various relaxation processes.

After e-ph thermalization, there are still excess electrons (holes) remaining in the conduction (valence) band. The intermediate relaxation process with a characteristic relaxation time of *τ*_2_~7–9 ps can therefore be associated with e-h recombination between the conduction and valence bands. In principle, such recombination may proceed via a radiative pathway, in which an electron in the conduction band can recombine with a hole in the valence band by emitting a photon. However, this scenario is unlikely for *τ*_2_ in NiTe_2_, since radiative recombination generally occurs on much longer timescales, typically on the order of several nanoseconds [41]. Alternatively, e-h recombination can occur through a three-body Auger process [42,43,44], where an electron and a hole recombine and transfer the released energy to a third carrier. Nevertheless, this mechanism is not supported by the pump fluence dependence of *τ*_2_ observed in our experiments. In Auger recombination, the relaxation rate scales with the third power of the carrier density, given by $dn/dt\propto -Cn^{3}$ [45,46]. This implies that Auger recombination would lead to a strong fluence-dependent recombination lifetime, in clear contradiction to the near-constant *τ*_2_ observed here. Finally, considering that NiTe_2_ is a semimetal with energy band overlap, and its conduction and valence band extrema are located at different points in the Brillouin zone. Consequently, direct interband e-h recombination requires additional momentum to satisfy momentum conservation, which can be supplied by phonons. We therefore attribute *τ*_2_ to phonon-assisted e-h recombination between momentum-mismatched electron and hole pockets, as schematically illustrated in Figure 3. As e-h recombination proceeds, the carrier population in the conduction band decreases, thereby weakening the band-filling effect and leading to the recovery of the bleaching signal. This behavior is consistent with the positive contribution of *τ*_2_ to the Δ*R*/*R*_0_ response shown in Figure 2d.

Following the establishment of quasi-thermal equilibrium between the hot electrons and hot optical phonons (*τ*_1_), the subsequent dynamics are dominated by energy redistribution among phonon subsystems through phonon–phonon (ph-ph) scattering. Therefore, the process *τ*_3_, with a time constant of ~20–30 ps, can be attributed to the anharmonic energy transfer from hot optical phonons to other uncoupled phonons (primarily lower energy acoustic phonon branch), similar to previous experimental and theoretical studies on Dirac materials [47,48,49]. It is noted that *τ*_3_ exhibits a weak pump fluence dependence, which reflects that the ph-ph scattering rate is nearly constant over the applied fluence range. Finally, the offset term C is usually associated with spatial heat diffusion out of the excitation volume on a timescale much longer than our measurement window (~200 ps) [37,38,47]. The thermal spot created by pump pulses eventually cools down by transferring heat to the surroundings, thus the long-lived offset component can be ascribed to a spatial flow of heat carried by acoustic phonons. Given that the size of the NiTe_2_ microcrystal studied here is comparable to the pump spot, lateral heat flow is negligible. Considering that the optical penetration depth of ~10 nm [50] is smaller than the thickness of NiTe_2_ flakes, heat diffusion occurs predominantly via longitudinal diffusion into the bulk.

**Figure 4:**
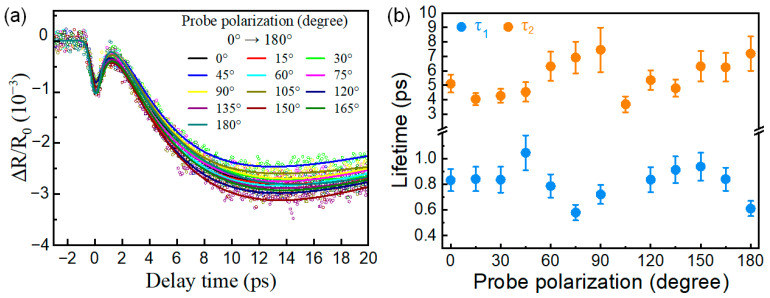
Probe polarization dependence of the ultrafast dynamics in NiTe_2_ microcrystals. (**a**) Transient reflectivity traces measured at probe polarization angles from 0° to 180° (where 0° is defined as the direction orthogonal to the pump polarization). The circular symbols represent the experimentally measured data, while the solid lines correspond to the fitting results. (**b**) Extracted relaxation times *τ*_1_ and *τ*_2_ at various probe polarization angles.

Additionally, we investigated the transient in-plane isotropy of NiTe_2_ microcrystals. We fixed the pump beam polarization and tuned the probe polarization angle with a half-wave plate. Figure 4a shows the transient reflectivity trace Δ*R*/*R*_0_ measured at various probe polarization angles from 0° to 180°, where 0° is defined as orthogonal to the pump polarization. It can be seen that the transient signals do not vary noticeably with the probe polarization angle, indicating that the underlying dynamics are insensitive to the polarization state of the probe beam. In Figure 4b, the extracted *τ*_1_ and *τ*_2_ relaxation times as a function of polarization angle do not exhibit significant variations within the experimental error. These results indicate that the ultrafast optical response of NiTe_2_ is nearly in-plane isotropic under the current pump-probe configuration, which is in significant contrast to many typical topological semimetals, where they often exhibit strongly anisotropic photocarriers and phonon dynamics [51,52,53,54]. Such isotropic behavior is beneficial for the design of polarization-insensitive optoelectronic devices, enabling robust performance under arbitrary light polarization.

It is worth mentioning that Cheng et al. [27] previously performed a degenerate 800 nm OPOP study on a bulk NiTe_2_ single crystal (~1 mm) employing a high-repetition-rate setup (80 MHz), identifying sub-picosecond and few-picosecond timescale relaxation components attributed to e-ph scattering and phonon-assisted e-h recombination, respectively. Our study on NiTe_2_ microcrystals not only reproduced these results (*τ*_1_ and *τ*_2_), but also extended the analysis to the slower recovery dynamics in detail, including ph-ph coupling (*τ*_3_) and longitudinal thermal diffusion (offset term C). In addition to mapping the full relaxation processes, we examined the transient in-plane isotropy dynamics of the NiTe_2_ microcrystals. These findings demonstrate that bulk-derived ultrafast relaxation mechanisms remain valid in device-compatible NiTe_2_ microcrystals, underscoring their relevance for integrated optoelectronic applications. Looking forward, recent studies have demonstrated the capability of the fractional description in analyzing complex photoconductive and nonlinear optical behaviors in micro- and nanomaterials [55]. In light of this, applying such a model to NiTe_2_, when combined with ultrafast spectroscopy, could pave the way for the development of optoelectronic circuits and all-optical signal processing in NiTe_2_.

## 4. Conclusions

We conducted a comprehensive investigation of ultrafast carrier and phonon dynamics in microcrystalline NiTe_2_ using time-resolved OPOP spectroscopy. The complete relaxation dynamics are resolved, encompassing sub-picosecond e-ph scattering, phonon-assisted e-h recombination, anharmonic decay of optical phonons into acoustic phonons, and eventual heat diffusion into the bulk. Polarization-dependent measurements further reveal a nearly isotropic ultrafast optical response, underscoring the robustness of NiTe_2_ for polarization-insensitive device applications. Our results unravel and validate the microscopic relaxation mechanisms in Dirac semimetal NiTe_2_, thereby laying a foundation for the development of NiTe_2_-based micrometer-scale, ultrafast optoelectronics devices.

## Data Availability

The original contributions presented in this study are included in the article. Further inquiries can be directed to the corresponding authors.
